# Supplementary material for: A Risk Prediction Model and Risk Score of SARS-CoV-2 Infection Following Healthcare-Related Exposure
Source: Trop Med Infect Dis. 2022 Sep 14;7(9):248. doi: 10.3390/tropicalmed7090248 (PMC9505412; doi:10.3390/tropicalmed7090248)
Supplement: Supplementary file 1 [file tropicalmed-07-00248-s001.zip › tropicalmed-1911094-supplementary.pdf]

Supplementary Table S1 Exposure characteristics-based risk classification

**Exposure detail**		**Duration of contact (Cumulative within 24 hours)**	**Contact risk category**
**PPE of contact personnel and exposure distance**	**PPE of Index cases**		
- Surgical mask with eye protection or<br>- Surgical mask with distance >1 meter	Surgical or cloth mask	Any	Insignificant
	No mask	≤15 minutes	Low
	No mask	>15 minutes	Moderate
- Surgical mask with distance ≤1 meter without eye protection	Surgical or cloth mask	≤15 minutes	Low
	Surgical or cloth mask	>15 minutes	Moderate
	No mask	≤15 minutes	Moderate
	No mask	>15 minutes	High
- Contact personnel not wearing a mask<br>- Improper PPE during an aerosol-generating procedure (at least N95 respirator and eye protection)<br>- Direct contacts of mucosa with the aerodigestive secretion of index case without proper hand hygiene or sharing food with the same utensils<br>- Eating together without a barrier board<br>- Close contact in the same vehicle		Any	High
- Indirect contacts with fomite of index case without proper hand hygiene		Any	Moderate
- Eating together with a barrier board		>15 minutes	Moderate
- Eating together with a barrier board without talking or sharing food		≤15 minutes	Low

Supplementary Table S2 Management of contact hospital workers in terms of guided testing and quarantine duration based on contact risk and vaccination history

Contact risk category	Immunization history*	Guidance of testing date after last known contact	Quarantine duration (days)
High	Immunized	Immediate, day #7, day #14	7
	Unimmunized		14
Moderate	Immunized	Day #7, day #14	No
	Unimmunized		7–14**
Low	Any	Once during day #7–14	No

* See Supplementary Table S3

** Depend on the judgement of the division chief according to the extent of staff shortage

Supplementary Table S3 Definition of immunization during the study period

Time	Factors determine immunity		
	**Number of total vaccine doses**	**Vaccine regimens**	**Minimum duration (days) from last shot to the date of exposure**
Before August 27, 2021	2	CoronaVac	14
	1	ChAdOx-1	28
	OR previous COVID-19 infection within the past 90 days		
August 27, 2021, and later	3	2 of CoronaVac + booster with mRNA or ChAdOx-1	14
	3	Inactivated vaccine	28
	2	2 of mRNA or<br>2 ChAdOx-1 or<br>Inactivated vaccine+ChAdOx-1 or<br>ChAdOx-1+ mRNA	14
	OR previous COVID-19 infection within the past 90 days		

Supplementary Table S4 Vaccine regimen potency grouping, adapted from Thai COVID-19 vaccination guidelines for a booster shot from the Ministry of Public Health as of December 2021.

Potency group	Vaccination history		Recommended booster duration after last shot
	**Doses**	**Regimens**	
Low	1–3	Inactivated vaccine (CoronaVac or BBIBP)	4 weeks
	1	ChAdOx-1 or mRNA	4–12 weeks
Moderate	3	2 inactivated vaccines plus 1 ChAdOx-1 or mRNA	3 months
	2	ChAdOx-1	3 months

	2	Inactivated vaccine plus ChAdOx-1 or mRNA	3 months
High	3 or more	Contain at least ChAdOx-1 plus mRNA or 2 mRNA	Booster not recommended
	2	mRNA (BNT162b2 or mRNA-1273)	6 months
	2	ChAdOx-1 plus mRNA	6 months

Supplementary Table S5 Parallel analysis of variables associated with SARS-CoV-2 infection using exposure risk category, the final logistic model

**Risk factor**	**β**	**Adjusted odds Ratio (95% CI)**	**P-value**
The highest education attainment			0.000
Primary or secondary school (reference)			
Undergraduate (associate's or bachelor's)	−0.66	0.52 (0.4–0.67)	0.000
Postgraduate (master's or doctoral)	−1.25	0.29 (0.17–0.48)	0.000
Exposure risk category			0.000
Low risk (reference)			
Moderate risk	0.95	2.57 (1.44–4.59)	0.001
High risk	2.14	8.53 (5.16–14.1)	0.000
Insignificant risk with symptom(s) or reasons for RT-PCR	0.89	2.43 (1.23–4.78)	0.010
Fever or other COVID-19 related symptoms	1.67	5.31 (4.10–6.88)	0.000
Vaccination group			0.000
Unvaccinated (reference)			
Low potency	−1.08	0.34 (0.2–0.59)	0.000
Moderate potency	−1.52	0.22 (0.12–0.39)	0.000
High potency	−2.68	0.07 (0.01–0.53)	0.010
Constant	−3.68		0.000

Supplementary Figure S1 Daily number and cumulative percentage of occupational exposures among healthcare personnel in the study, 15 September to 31 December 2021

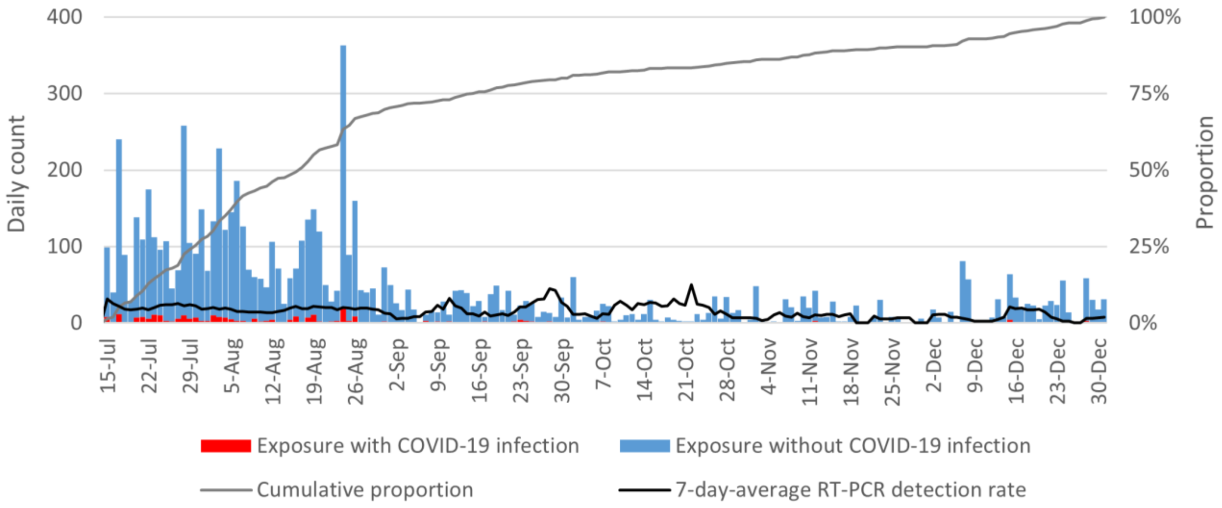

Supplementary Figure S2 Distribution of SARS-CoV-2 PCR assay detection day after last known exposure

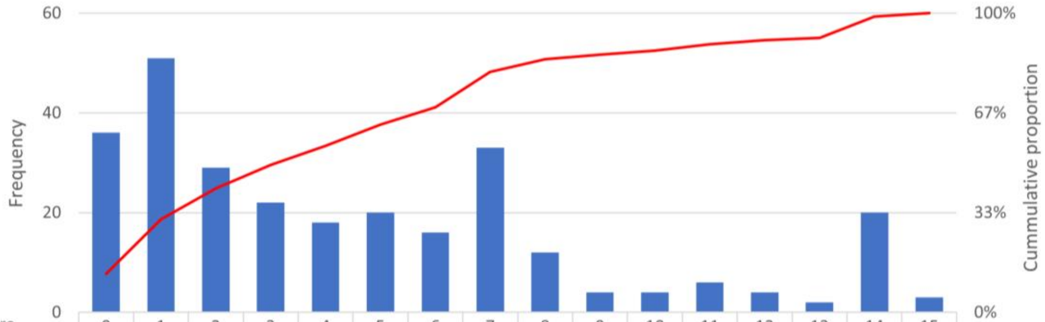

Day after last exposure	0	1	2	3	4	5	6	7	8	9	10	11	12	13	14	15
Frequency	36	51	29	22	18	20	16	33	12	4	4	6	4	2	20	3
Cummulative proportion	13%	31%	41%	49%	56%	63%	69%	80%	85%	86%	88%	90%	91%	92%	99%	100%
